# 

**Published:** 1894-02-10

**Authors:** 


PRICE TWOPENCE.

No. 385, Vol. XV.—Feb. 10th, 1894.

[Registered at the General Post Office as a Newspaper for transmission in the United Kingdom and Abroad.]

WITH EXTRA NURSING SUPPLEMENT.

Per Annum, 8s. 6d., post free.

Monthly Parts, 6d.; post free, 8d.

Ditto per Annum, 8s., post free.

### THE HOSPITAL

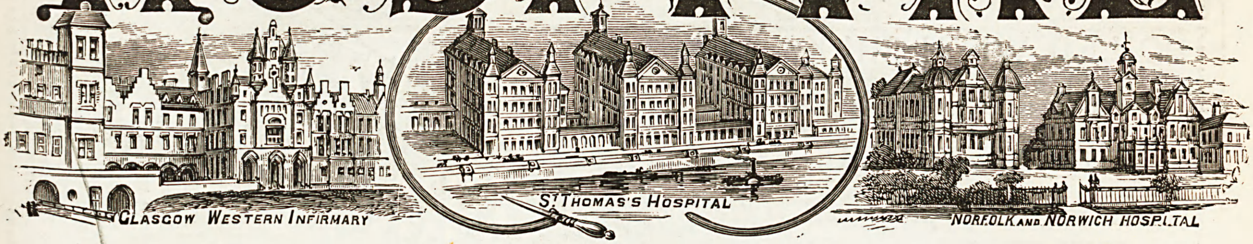

No. 385. Vol. XV. WITH EXTRA NURSING SUPPLEMENT. SATURDAY, FEBRUARY 10TH, 1894.

